# Observations on the Nature and Treatment of Angina Pectoris

**Published:** 1894-01

**Authors:** 


### OBSERVATIONS ON THE NATURE AND TREATMENT OF ANGINA PECTORIS.

J. Burney Yeo, (*Boston Medical and Surgical Journal,*) after discussing the known modes of causation of attacks of angina pectoris, summarizes the indications for treatment as follows:

1. To maintain or improve, when defective, the general nutrition; to avoid all strain, physical and emotional; and so to relieve cardiac feebleness and excessive effort.

2. To relieve dyspeptic conditions and flatulent or fecal distension of the stomach and intestines.

3. To forbid the habitual consumption of agents which may exercise a toxic action on the heart, such as tea, coffee, tobacco, alcohol, etc., or that may introduce or develop toxines in the alimentary canal.

4. To avoid and remove all gouty and other blood contaminations.

5. To give such tonic remedies as may improve the cardiac tone and lessen existing tendency to cardiovascular degeneration.

6. To relieve the paroxysmal attacks by sedatives and stimulants.

1. Under the first indication, Dr. Yeo speaks of the necessity of careful attention to hygienic measures; the life of the patient must be one of complete repose of mind and body—a repose alternated with gentle exercise. Life in the open air and in a mild climate is also beneficial. The diet must be carefully looked after, and sometimes be almost exclusively of milk. If the digestive powers are greatly weakened, it may be necessary to have recourse to pre-digested foods, or to give with the food some artificial digestive agent. The writer mentions a number of dishes that may be easily assimilated, and then goes on to say the physician should see that a sufficient quantity of water is taken, both for eliminative and assimilative purposes, a point often overlooked.

2. The coëxistence of dyspeptic states must be treated in accordance with general principles—an alkaline bitter stomachic, composed of sodium bicarbonate, nux vomica, and columbo, an hour before the two principal meals, will be found valuable. Regular evacuation of the bowels of fecal accumulations is most essential—checking as it does the formation of injurious toxines in the intestines, eliminating waste substances, and relieving abdominal disturbance. Dr. Yeo here suggests various remedies as aperients.

3. This indication is also an important one, for certain of the slighter forms of angina are no doubt dependent on, and the more serious forms may be provoked by, the habitual use of certain substances which come, in course of time, to exercise a toxic action on the heart. The action of these toxic agents is all the more subtle because they may be taken for many years without apparently producing any injurious effect. This is particularly the case with tobacco, the toxic effects of which on the heart are often delayed until, or even after middle age, when they will perhaps somewhat suddenly make themselves felt. The toxic and degenerating influence of alcohol falls upon different organs in different individuals ; but whenever anginal symptoms arise, complete abstinence from alcohol should be insisted on or its very sparing use in very dilute form. Tea and coffee are often provocative of the slighter manifestations of cardiac pain and discomfort, and they are prone to be aggravated by any emotional disturbance. All these toxic agents must be forbidden so long as any tendency to anginal attacks exists.

4. The next indication is to remove and avoid all gouty and other blood contamination. The importance of elimination in the treatment of angina pectoris is universally admitted. When renal elimination is defective from the coëxistence of renal degeneration, the bowels and the skin must be acted on. When the kidneys are sound, the free use of pure water, or some suitable mineral water having some slight stimulating action on the kidneys, may avoid the necessity of free purgation ; but, in all cases, a thorough daily evacuation of the bowels should be procured, and free action of the skin should be maintained by warm baths and frictions. In gouty cases, and in all cases of defective elimination, the diet should be sufficient, but careful and spare, avoiding all excess. Animal food should be taken only in great moderation, and fresh vegetables and fruit carefully prepared, and cooked so as to be made easy of digestion, should take its place. All alcoholic stimulant should be avoided, and, if acceptable to the patient, a few weeks of exclusive milk diet may be advantageous.

5. Dr. Yeo considers next the medicinal treatment of these cases, and first, the appropriate treatment in the intervals—that is, of the constitutional condition underlying the paroxysmal attacks. He points out in certain cases the value of the milder preparations of iron, combined with small doses of digitalis, in other cases of the greater value of arsenic ; the usefulness of strychnine as a cardiac tonic, of potassium iodide, when angina pectoris is associated, with obvious signs of cardiac vascular degeneration and of the gouty state, etc.

6. Those who see in the causation of the anginal paroxysm the predominating influence of vaso-motor spasm, consider that the main indication for the relief of the paroxysm is to administer medicinal agents which are known to have the power of relaxing the arterioles ; they therefore advocate the use of the nitrites, such as the nitrite of amyl, nitro-glycerine, and sodium nitrite. That these agents do relieve the paroxysm in many cases of angina, is certain ; that they do so wholly by their action as vaso-dilators, is extremely doubtful. In the first place, they are capable of relieving the anginal attack when there is no certain evidence of the existence of vaso-motor spasm. Dr. Douglas Powell says he has found them "far more reliable in the graver cardiac cases than in the purer vaso-motory," but it is in the latter that they should prove most efficacious if the prevailing theory of their action were true. Balfour and Grainger Stewart both believe that they act as direct analgesic agents, and that they have the power of relieving pain in other as well as in cardiac neuralgias, independently of their relaxing action on the bloodvessels ; and this view Dr. Yeo adopts as the most consistent with the clinical history of the disease.

The effect of sodium nitrite is said to be more lasting than that of nitrite of amyl or nitro-glycerine. It is given in tablets of $2\frac{1}{2}$ grains ; one to four of these may be given for a dose. At the onset of an attack, in addition to the inhalation of the nitrite of amyl, which, owing to the rapidity of its action, is the most suitable remedy to start with, some warm diffusable stimulant may be given, such as thirty minims of sulphuric ether or a drachm of nitrous ether, with a drachm of sal volatile, or a little brandy in an ounce or two of peppermint water. Feet and hands, if cold, may be placed in hot water. Balfour has been disappointed in action of nitro-glycerine, and prefers inhalations of nitrite of amyl ; and when these fail—as they often will—he resorts unhesitatingly to chloroform inhalations ; sulphuric ether may be used, but it is not rapid enough. Chloroform may be poured on a sponge in a smelling bottle, and the patient is told to breathe it through his nose as deeply as possible. "In this way relief is obtained in a few seconds, and so soon as the narcotic influence is produced, the smelling bottle drops, and with it rolls away all risk of any overdose." In severe and protracted attacks it may be necessary to have recourse to hypodermic injections of morphine.

